# IVUS-Guided Pulmonary Artery Hemodynamic Sensor Implantation: A Novel Contrast-Sparing Strategy

**DOI:** 10.1016/j.jaccas.2025.105227

**Published:** 2025-09-24

**Authors:** Nishant Trivedi, Shreya Srivastava, Sydney Arus, Andrew Persits, Kristen Stawiarski, Rohit Maini, John Kassotis

**Affiliations:** a Department of Cardiology, Zucker School of Medicine at Hofstra/Northwell, Peconic Bay Medical Center, Riverhead, New York, USA; b Department of Internal Medicine, Zucker School of Medicine at Hofstra/Northwell at NS/LIJ, Uniondale, New York, USA; c State University of New York, New Paltz, New York, USA; d Department of Cardiology, Zucker School of Medicine at Hofstra/Northwell at NS/LIJ, Uniondale, New York, USA

**Keywords:** CardioMEMS, chronic kidney disease, contrast, heart failure, intravascular ultrasound, nephrotoxic

## Abstract

**Objective:**

This case report describes a novel insertion technique of a hemodynamic sensor (CardioMEMS), avoiding the need for intravenous contrast in patients with baseline renal dysfunction.

**Key Steps:**

Intravascular ultrasound (IVUS) was employed to both visualize and direct the placement of the CardioMEMS. IVUS, along with fluoroscopy, were used to identify the left pulmonary artery as well as guide and confirm sensor placement.

**Potential Pitfalls:**

The use of IVUS requires the operator to be familiar with identifying the appropriate target of the left pulmonary artery on ultrasound imaging. In addition, the introduction of IVUS in complex anatomy or a heavily calcified vessel may increase the risk of complications, including vessel perforation, dissection, and thrombosis.

**Take-Home Messages:**

IVUS is a feasible technique for CardioMEMS placement, minimizing the need for contrast. This is of particular importance in patients with heart failure and renal dysfunction, placing them at high risk for progression to renal failure.

## Graphical Abstract

**Figure:**
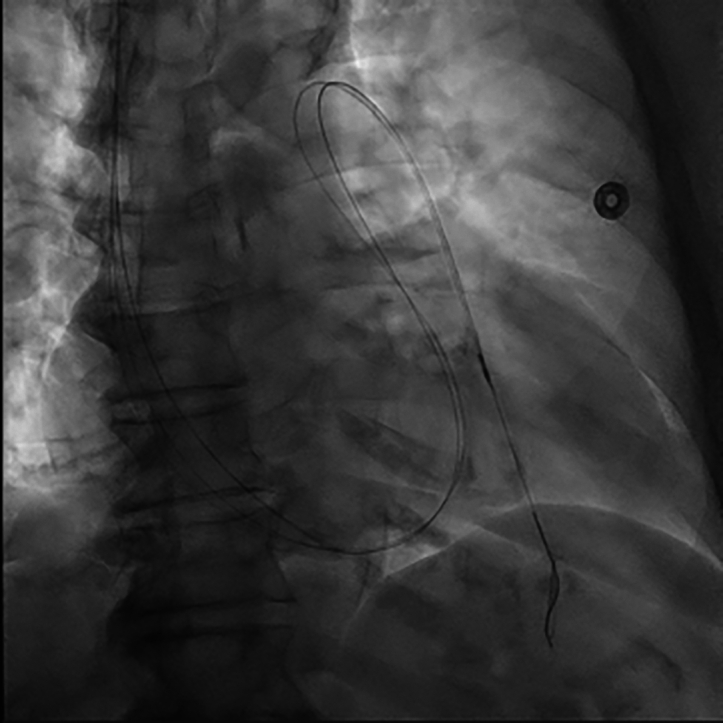

Heart failure is a global epidemic that affects more than 64 million people worldwide, including nearly 6.5 million Americans over the age of 20, and it requires a growing arsenal of therapies for its effective management.^1^ A significant metric in assessing how effectively patients with heart failure are managed is hospital readmissions. Hospital readmissions are not only a heavy financial burden on the health care system, it also correlates with patient mortality. Since its initial approval by the Food & Drug Administration in 2014, the CardioMEMS HF System (Abbott) has proven to be a powerful asset in the management of heart failure, specifically in patients with NYHA functional class III heart failure. The CHAMPION trial demonstrated that the use of CardioMEMS resulted in a reduction of hospital readmissions by 40% among these patients.^1^^,^^2^ As of January 2025, the Centers for Medicare & Medicaid, under their Coverage with Evidence Development framework, expanded coverage of implantable pulmonary artery pressure sensors in patients with heart failure who meet the criteria specified by the national coverage determination. These criteria are focused on patient symptoms and quality of life and are irrespective of left ventricular ejection fraction.^3^

Most commonly, this hemodynamic sensor is implanted via a transvenous transcatheter approach and is advanced fluoroscopically into a segment of the left pulmonary artery (LPA). Angiography with iodinated contrast is employed for targeted site selection. The use of angiography and contrast poses a risk of progression to renal failure in patients with chronic kidney disease (CKD). This case highlights a novel, contrast-sparing strategy for CardioMEMS implantation that relies on intravascular ultrasound (IVUS) for both deployment and site selection. This strategy will prove beneficial for patients with renal dysfunction or other contraindications to contrast exposure. In addition, use of the internal jugular vein as an alternative to femoral venous access reduces the need for a prolonged supine recovery period.

## Case

We present a case of an 81-year-old woman with hypertensive heart disease and obesity complicated by heart failure with preserved ejection fraction (HFpEF) (left ventricular ejection fraction: 55%), non–insulin dependent diabetes mellitus, stage IV CKD (glomerular filtration rate: 18), moderate nonobstructive coronary artery disease, and chronic lower back pain that makes prolonged supine positioning a challenge. She had endured frequent hospitalizations over the previous calendar year for acutely decompensated HFpEF requiring intravenous diuresis. While standard CardioMEMS implantation uses minimal contrast (1-3 mL), the patient would only undergo the procedure if it were performed without contrast, fearful of even a minimal risk of nephrotoxicity and need for dialysis.

## Procedure

After obtaining informed consent, the patient was brought to the cardiac catheterization laboratory, where she was prepared and draped in the usual sterile manner. The procedure was performed by an interventional cardiologist, but it is also often performed by interventional heart failure specialists. It was decided to obtain vascular access via the right internal jugular (RIJ) vein. This was the preferred access strategy to minimize the time that the patient needed to be supine postprocedure, compared to the more traditional femoral access. Using an ultrasound-guided modified Seldinger technique, a 7-F, 10-cm Pinnacle sheath (Terumo) was inserted into the RIJ, aspirated, and flushed. This sheath was exchanged for a 12-F, 12-cm Performer sheath (Cook Medical), under fluoroscopic guidance, over a 0.035-inch J-tipped guidewire. A 7-F Swan-Ganz pulmonary artery catheter (PAC) (Edwards Life Sciences) was advanced under fluoroscopic guidance across the right atrium, right ventricle, and pulmonary artery. Pressure measurements were recorded, and the cardiac output was calculated.

**Figure 1:**
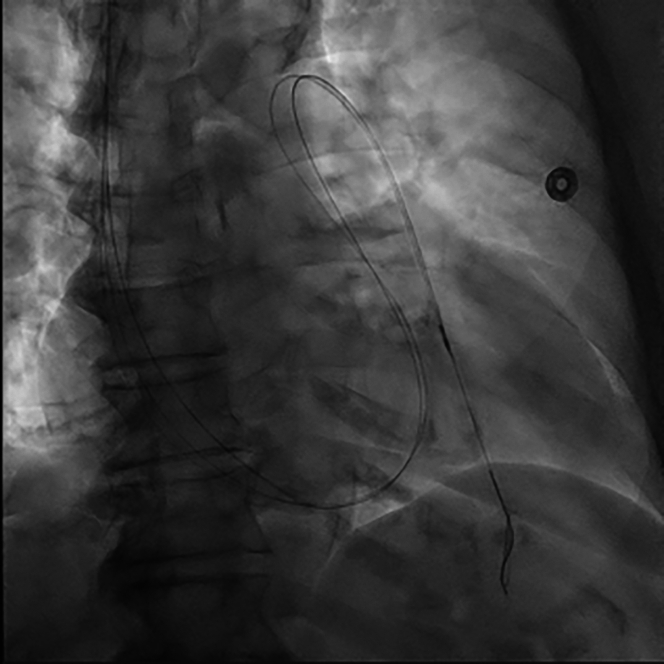
IVUS Catheter Advancing Into Distal LPA Target, With a Second Adjacent “Buddy Wire” Conferring Additional Support IVUS = intravascular ultrasound; LPA = left pulmonary artery.

**Figure 2:**
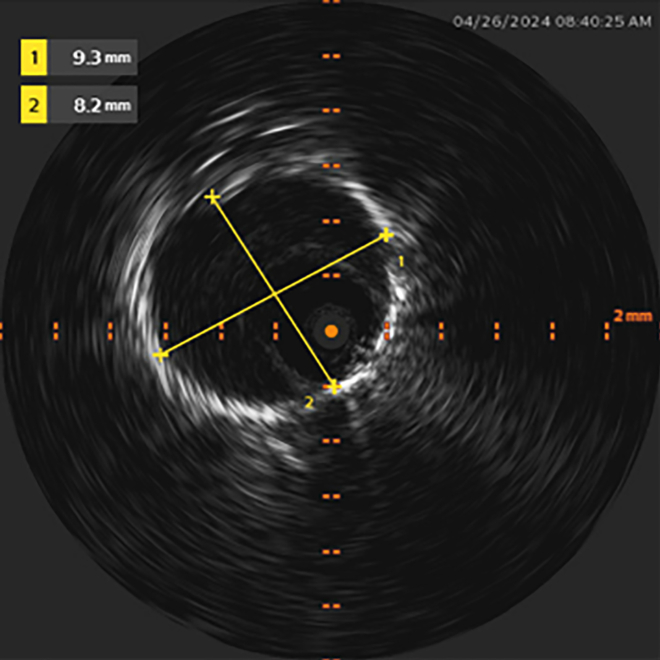
Measurement of LPA Segment Diameter Using IVUS IVUS = intravascular ultrasound; LPA = left pulmonary artery.

PAC engagement of the LPA was facilitated by a Roadrunner 0.018-inch wire (Cook Medical), over which the PAC was advanced. The Roadrunner wire was carefully inserted into a posteriorly directed segment of the LPA, guided by left anterior oblique and anteroposterior fluoroscopic projections. This technique is in line with the current standard of care. Alongside the Roadrunner wire, a 260-cm 0.018-inch V-18 guidewire (Boston Scientific) was advanced through the PAC distal port lumen, at which point the PAC was removed over both wires (Figure 1). An 0.018-inch Visions PV IVUS catheter (Philips) permitted clear visualization of lumen patency and diameter as it was advanced over the V-18 guidewire into the LPA (Figure 2). The use of the Roadrunner wire alongside the V-18 guidewire allowed for increased stabilization of the IVUS catheter. This is referred to as the “buddy wire” technique. Pullback of the IVUS catheter allowed for the identification of an LPA segment with optimal lumen size proximally and distally. The proximal and distal boundaries identified on IVUS were bookmarked and “co-registered” with corresponding fluoroscopic images of the IVUS catheter's position in relation to bony landmarks. Leaving the Roadrunner guidewire behind, the IVUS catheter and the V-18 guidewire were removed.

**Figure 3:**
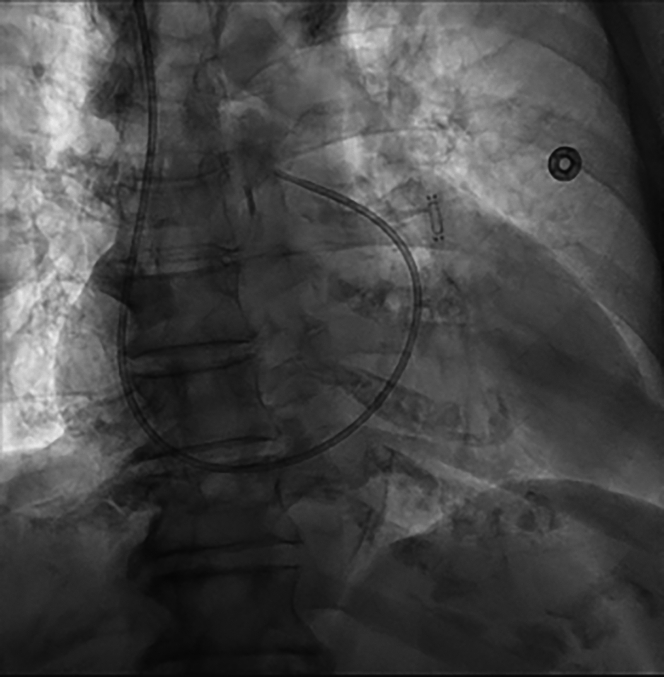
Successfully Deployed CardioMEMS Device; 7-F Pulmonary Artery Catheter in Place for Device Calibration

With the bookmarked fluoroscopic images serving as proximal and distal reference points, the CardioMEMS device was prepared in the usual fashion and was advanced over the Roadrunner wire to the target LPA segment and deployed successfully in the desired orientation (Figure 3). The delivery system was then removed. The PAC was readvanced into the LPA for postimplant calibration, after which it was removed from the patient. A single figure-of-eight suture provided hemostasis after removal of the Roadrunner wire and 12-F sheath. The length of the procedure was comparable to the time of traditional CardioMEMS implantation (approximately 30 minutes). The patient was then transferred to the recovery area in stable condition and was subsequently discharged home.

## Discussion

In the ongoing effort to mitigate the challenges of a global heart failure epidemic, it is important to consider comorbid diseases and the necessity for innovative treatment options. Up to 50% of patients who have experienced heart failure have concomitant CKD.^3^ Patients with renal dysfunction are particularly susceptible to complications associated with the use of iodinated contrast dye. There is a reported 12.8% incidence of acute kidney injury after coronary angiography, with a concomitant 20.2% mortality rate.^4^ Currently, for standard implantation of the CardioMEMS device, angiography is performed with contrast dye to visualize blood vessels and guide catheter and device implantation.

IVUS-guided placement of the CardioMEMS device without contrast-mediated angiography is a novel approach, performed in this scenario at the request and discretion of the patient. It was a successful method of guiding CardioMEMS implantation while eliminating the risk of complications of contrast dye in a patient with CKD. A CardioMEMS insertion uses minimal contrast, and contrast-mediated allergies can be pretreated, thus we anticipate this approach will be favored by patients such as ours who have CKD and are fearful of progressing to dialysis. This approach can potentially be a standard method of CardioMEMS insertion in patients with comorbid renal dysfunction or other conflicting conditions, but it warrants more research. The use of the hydrophilic V18 wire can increase the risk perforation when navigating the vasculature, however in our case the wire was used to support the catheter, not for navigation, thereby reducing the risk of perforation. It is our belief that this approach can be modified to rely solely on IVUS without the use of fluoroscopic images, sparing both the implanter and patients of any radiation exposure.

These findings may have implications for performing other cardiac interventions, either with IVUS alone or in conjunction with angiography. A meta-analysis of 30,814 angiography examinations, in which IVUS-guided treatment was used for 4,991 of them, found that IVUS is associated with improved survival rate for patients undergoing percutaneous coronary intervention and diagnostic coronary angiography.^5^ This is likely owing to the increased visual accuracy attained with the IVUS leading to more appropriate interventions.^5^

Cost analysis comparing use of angiography versus IVUS for CardioMEMS placement has yet to be demonstrated. However, with regard to percutaneous coronary intervention, given the improved outcomes and reduced adverse effects, IVUS has been shown to have overall equivalent cost-effectiveness to angiography.^6^ Implementing IVUS into common practice for CardioMEMS placement may yield similar findings.

## Conclusions

This case demonstrates that an IVUS-guided CardioMEMS implantation approach was a safe and straightforward alternative to traditional implantation using contrast angiography, reducing the risk of acute kidney injury. Further experience is needed to determine if an IVUS-only approach is universally feasible, reducing radiation exposure. By minimizing risk and discomfort, with the expanded implantation coverage, more patients will reap the benefits of pulmonary artery pressure sensor implantation. This has the potential to further improve quality of life by further reducing hospital admissions in patients with heart failure.

An RIJ approach conferred a greater ease of insertion compared to a femoral approach. This was ergonomically comfortable for the patient and expedited a same-day discharge. If stability of wire positioning during equipment delivery and removal is lacking (commonly encountered with severe right heart dilation), the “buddy wire” technique confers additional structural support by adjacent placement of the second guidewire.

## Funding Support and Author Disclosures

The authors have reported that they have no relationships relevant to the contents of this paper to disclose.

**Take-Home Message**

• IVUS is a feasible method for CardioMEMS placement that minimizes the need for contrast in patients with chronic kidney disease. The reduced adverse effects associated with IVUS may lead to fewer readmissions, thus demonstrating the appropriate cost-effectiveness of this methodology.

**Equipment List:** 

7-F × 10-cm Pinnacle Sheath (Terumo)
11-F × 10-cm Pinnacle Sheath (Terumo)
12-F × 12-cm Performer Sheath (Cook Medical)
CardioMEMS HF System (Abbott)
Visions PV 0.018-inch digital IVUS catheter (Philips)
Roadrunner 0.018-inch curved tip wire (Cook Medical) – for MEMS implant
Platinum Plus 0.018-inch 260-cm wire (Boston Scientific) – for IVUS
7-F × 110-cm Swan-Ganz pulmonary artery catheter (Edwards Life Sciences)
